# Zebrafish as a Suitable Model for Utilizing the Bioactivity of Coumarins and Coumarin-Based Compounds

**DOI:** 10.3390/ijms26041444

**Published:** 2025-02-08

**Authors:** Joanna Lachowicz-Radulska, Jarosław Widelski, Filip Nowaczyński, Anna Serefko, Jan Sobczyński, Agnieszka Ludwiczuk, Natalia Kasica, Aleksandra Szopa

**Affiliations:** 1 Department of Clinical Pharmacy and Pharmaceutical Care, Medical University of Lublin, 7 Chodźki Street, 20-093 Lublin, Poland; joanna.lachowicz@umlub.pl (J.L.-R.); filip.nowaczynski@umlub.pl (F.N.); anna.serefko@umlub.pl (A.S.); jan.sobczynski@umlub.pl (J.S.); 2 Department of Pharmacognosy with Medicinal Plants Garden, Medical University of Lublin, 1 Chodźki Street, 20-093 Lublin, Poland; jaroslaw.widelski@umlub.pl (J.W.); agnieszka.ludwiczuk@umlub.pl (A.L.); 3 Department of Animal Anatomy, Faculty of Veterinary Medicine, University of Warmia and Mazury in Olsztyn, 10-719 Olsztyn, Poland; natalia.kasica@uwm.edu.pl

**Keywords:** coumarin, coumarin-based compounds, zebrafish model, bioactivity of coumarins

## Abstract

The aim of this review is to summarize the current knowledge on the use of coumarin-derived compounds in the zebrafish (*Danio rerio*) model. Coumarins, a class of naturally occurring compounds with diverse biological activities, including compounds such as coumarin, angelicin, and warfarin, have attracted considerable attention in the study of potential therapeutic agents for cancer, central nervous system disorders, and infectious diseases. The capabilities of coumarins as active compounds have led to synthesizing various derivatives with their own properties. While such variety is certainly promising, it is also cumbersome due to the large amount of research needed to find the most optimal compounds. The zebrafish model offers unique advantages for such studies, including high genetic and physiological homology to mammals, optical transparency of the embryos, and rapid developmental processes, facilitating the assessment of compound toxicity and underlying mechanisms of action. This review provides an in-depth analysis of the chemical properties of coumarins, their mechanisms of biological activity, and the results of previous studies evaluating the toxicity and efficacy of these compounds in zebrafish assays. The zebrafish model allows for a holistic assessment of the therapeutic potential of coumarin derivatives, offering valuable insights for advancing drug discovery and development.

## 1. Introduction

Coumarins are a diverse group of organic bioactive compounds belonging to benzopyrones (1,2-benzopyrones or 2H-1-benzopyran-2-ones) [1,2,3]. Its structure consists of two six-membered rings containing lactone carbonyl groups [4]. The most basic compound in this group is coumarin (2H-chromen-2-one, 2H-1-benzopyran-2-one), which is a chromenone with the keto group in the 2 position [5]. The chemical structure and nomenclature of exemplary coumarins are presented herewith (Table 1). The classification of coumarins is based on the criteria of chemical diversity and complexity. The various types of coumarin include simple coumarins (e.g., coumarin, umbelliferone, scopoletin, esculetin, scoparone, osthole), isocoumarins (e.g., isocoumarin, mellein, 4-hydroxymellein, monocerin, thunberginols), furanocoumarins (e.g., psoralen, angelicin, xanthotoxin, antoghenol, columbianedin), pyranocoumarins (e.g., grandivittin, inophyllum), biscoumarins (e.g., dicoumarol, thamnosin, bisparasin, hassmarin), and others, such as phenylcoumarins (e.g., 3-phenylocoumarin, isodispar) [6,7].

Coumarins are secondary metabolites that appear naturally in a large number of medical plant species, as well as in microorganisms (fungi and bacteria) and sea sponges. Coumarins are present in a variety of plant tissues, including flowers, fruits, leaves, roots, seeds, and stems [3,7,8,9]. A total of more than 1300 coumarins have been identified, exhibiting a wide distribution across *Angiospermae*, *Dicotyledoneae,* and *Monocotyledoneae* families [3]. A particularly high concentration of this phytochemicals was identified in tonka bean (*Coumarouna odorata*, *Dipteryx odoranta* Wild). Additionally, coumarins in high concentration are present in *Anthoxanthum odoratum* [10] and *Cinnamomum cassia* [11] but also in *Conioselinum* Fisch., *Ferula* L., *Heracleum* L., *Libanotis* L. *Prangos* Lindl., *Pachypleurum* Hoffm., and *Seseli* L. [4,12] (for a review, see [3,13]). Coumarins are predominantly synthesized in the fruits, leaves, and roots of plants [14], primarily via the phenylpropanoid pathway [15]. Their synthesis is initiated by deamination of the amino acid L-phenylalanine, a process catalyzed by the enzyme phenylalanine ammonia lyase (PAL), which results in the production of trans-cinnamic acid [16]. This acid is then converted into p-coumaric acid and o-hydroxycinnamic acid, which spontaneously cyclizes to form the basic coumarin structure [15]. However, it should be noted that the ultimate structure of coumarins is the consequence of subsequent specific modifications. These additional modifications, which may include methylation, hydroxylation, or glycosylation, are catalyzed by the relevant enzymes (for instance, O-methyltransferases and cytochromes P450). These modifications are influenced by the availability of enzymes and specific metabolic conditions within the plant organism in which they occur (for a review, see [1,15,16]).

**Table 1:** The chemical structure and nomenclature of exemplary coumarins (chemical structure created in ChemDraw 22.2.0).

Coumarin Name	Molecular Formula	Chemical Structure	Ref.
Simple coumarins			
Coumarin	C_9_H_6_O_2_	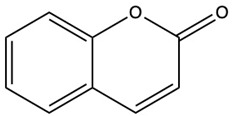	[5]
Umbelliferone (7-hydroxycoumarin)	C_9_H_6_O_3_	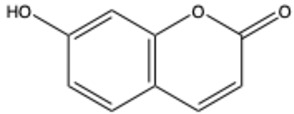	[17]
Esculetin (6,7-dihydroxycoumarin)	C_9_H_6_O_4_	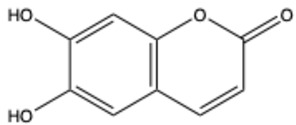	[18]
Scopoletin (6-methoxy-7-hydroxycoumarin)	C_10_H_8_O_4_	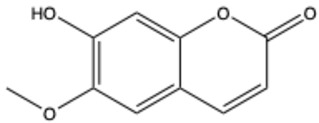	[19]
Scoparone (6,7-dimethoxycoumarin)	C_11_H_10_O_4_	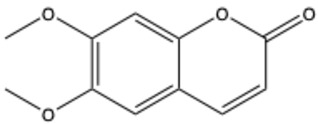	[20]
Osthole (7-methoxy-8-[3-methylpent-2-enyl]coumarin)	C_15_H_16_O_3_	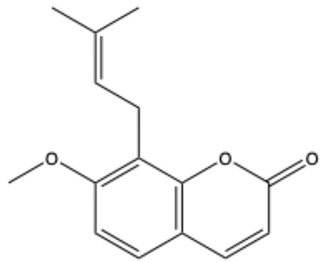	[21]
Warfarin	C_19_H_16_O_4_	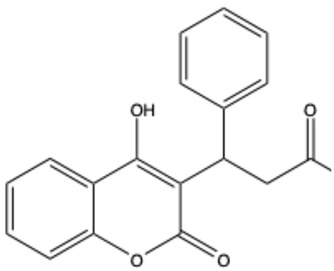	[22]
Isocoumarins			
Isocoumarin (benzopyran-1-one)	C_9_H_6_O_2_	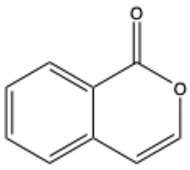	[23]
Mellein (3,4-dihydro-8-hydroxyisocoumarin)	C_10_H_10_O_3_	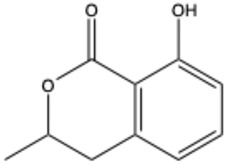	[24]
Monocerin	C_16_H_20_O_6_	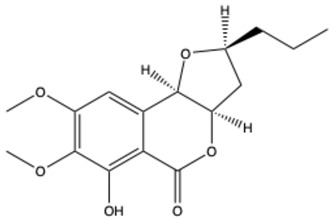	[25]
Furocoumarins			
Psoralen (furocoumarin)	C_11_H_6_O_3_	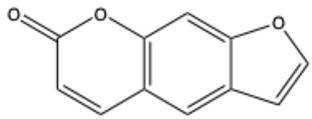	[26]
Angelicin (isopsoralen)	C_11_H_6_O_3_	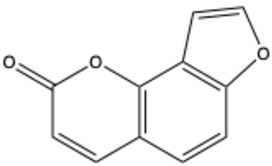	[27]
Xanthotoxin (8-methoxypsoralen)	C_12_H_8_O_4_	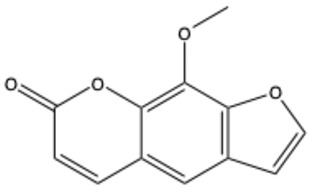	[28]
Pyranocoumarins			
Inophyllum A	C_25_H_24_O_5_	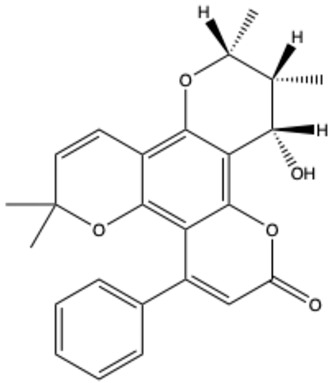	[29]
Biscoumarins			
Dicoumarol	C_19_H_12_O_6_	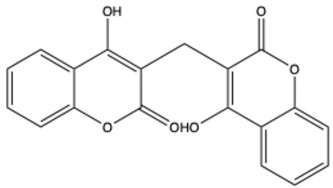	[30]
Thamnosin	C_30_H_28_O_6_	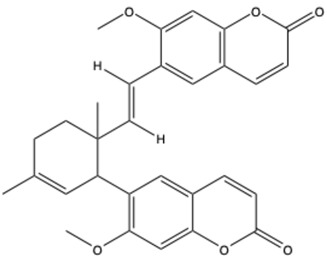	[31]
Phenylocoumarins			
3-Phenylcoumarin	C_15_H_10_O_2_	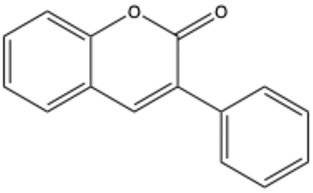	[32]
Isodispar B	C_20_H_18_O_5_	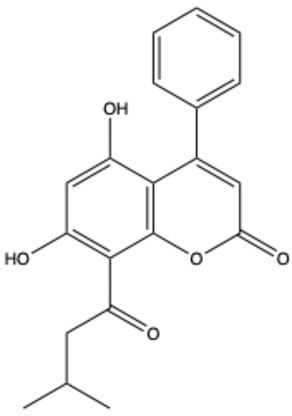	[33]

The use of coumarins in traditional practices across various cultures has spanned a considerable length of time, with the earliest documented evidence of using plants rich in coumarin content as medicinal plants dating back to ancient times [34,35]. Coumarins were first identified in 1820 by the German chemist Vogel, who extracted them from the tonka bean [36]. During the nineteenth and early twentieth centuries, scientists became increasingly interested in coumarins due to their distinctive aroma and taste. Coumarins were soon employed as an additive in perfumes, cosmetics, and tobacco products [37]. However, their potential biological activity and therapeutic properties remained largely unexplored until the discovery of dicoumarol, a natural anti-coagulant compound found in *Melilotus* spp. (spoiled sweet clover), in the 1940s [38]. This discovery prompted interest in coumarins as pharmacological agents [9,39]. As studies proceeded, researchers expanded their exploration of the coumarins’ biological and therapeutic properties, including anti-coagulant, anti-inflammatory, anti-microbial, anti-viral, anti-parasitic, anti-helmintic, molluscacidal, anti-oxidant, anti-diabetic, anti-hypertensive, anti-cancer, anti-proliferative, anti-convulsant, estrogenic, dermal photosensitizing, vasodilator, sedative, hypnotic, and analgesic properties (for a review, see [7,13,40]).

The advancement of the synthesis and modification of natural compounds has facilitated the development of a plethora of coumarin derivatives, thereby enabling the refinement of their biological effects. These derivatives have subsequently been investigated for a multitude of medical applications, including the treatment of cardiovascular diseases, neurological disorders, metabolic disorders, and cancer, as well as anti-microbial applications and much more [1,16,41,42]. The domain of research pertaining to coumarins and their derivatives persists as an active field of study. The manifold bioactive properties and prospective therapeutic applications of coumarins continue to be a source of significant interest. Contemporary research endeavors are investigating novel coumarin-based compounds for potential deployment in the management of diverse human diseases. The use of model organisms, such as the zebrafish (*Danio rerio*), has further advanced coumarin research, providing an effective system for screening and evaluating these compounds for both efficacy and safety.

Therefore, the aim of this review is to assess the suitability of zebrafish as an experimental model for the evaluation of the pharmacological activity of coumarins and their derivatives. In addition, this review will provide a basis for understanding the zebrafish as a valuable and versatile model for the pharmacological evaluation of coumarins, thus contributing to the wider application of this model in drug discovery and safety profiling.

## 2. Advantages and Disadvantages of Using the Zebrafish Model

The zebrafish (*Danio rerio*) is a small freshwater fish belonging to the ray-finned fish group of the family *Cyprinidae*. The zebrafish was first described by the Scottish physician Francis Hamilton [43]. The scientific name of the zebrafish, *Danio rerio*, is specific to this particular species, and it is unique to freshwater ecosystems within the geographic region of South Asia, particularly in the Indian subcontinent, Pakistan, Nepal, Bangladesh, Bhutan, and possibly Myanmar [44]. This fish has been a prominent subject of study in numerous research fields since the 1980s. It has become a model organism that has been at the forefront of several research areas since that time [45,46]. This can be attributed to the multitude of advantages that the zebrafish model offers to scientists, particularly within the domains of biology, pharmacology, pharmacy and medicine. Among these are the following.

(1) *Genetic similarity to humans.* A substantial proportion of zebrafish genes are identical to those found in humans, representing approximately 70% of the total [47], which makes them an invaluable model for the study of human disease and genetics.
(2) *Simplicity of genetic manipulation.* The zebrafish is a convenient model organism for genetic modification [48], as it can be modified using a variety of techniques [49], including CRISPR/Cas9 (clustered regularly interspaced short palindromic repeats) [50,51,52], morpholino injection [53], and transgenic approaches [54]. This makes it an ideal subject for the creation of models, which can be used to gain a deeper understanding of human diseases in a number of fields, including oncology, Alzheimer’s disease, immunology, diabetes, regenerative medicine, aging-related research, etc. [46].
(3) *High embryo yield and rapid reproduction rate.* The zebrafish is a highly prolific species, capable of producing hundreds of embryos per week. This reproductive rate allows for the utilization of large sample sizes in scientific experiments and high-throughput screening in drug discovery and toxicology [46].
(4) *Embryo/body transparency*. The transparency of zebrafish’s embryos and body throughout adulthood permits direct observation of organ development and cellular processes in real time [55]. This facilitates research into a range of topics, including tissue development, disease progression, and drug effects.
(5) *Development outside of the uterus*. The external development of zebrafish embryos outside of the uterus provides researchers with convenient access for a range of studies, particularly those requiring post-fertilization observation [46].
(6) *Fast development and a short life cycle.* The rapid development and short life cycle of zebrafish embryos provide an optimal environment for the evaluation of genetic and environmental interventions across generations. The embryos develop rapidly, with major organs forming within 24 h post-fertilization (hpf) and a fully functional body system within 120 hpf (5 days post-fertilization) [56]. This enables researchers to rapidly evaluate the impact of any intervention.
(7) *Cost-effectiveness*. In terms of cost-effectiveness, zebrafish offer distinct advantages over mammalian models. Their smaller size and simpler aquatic environment necessitate reduced space requirements and maintenance costs, making them a more cost-effective choice for long-term studies [46,57,58,59].
(8) *Ethical guidelines*. Zebrafish is categorized as a lower vertebrate, and research involving it is often subject to less stringent ethical guidelines than that conducted on rodents, for example. Additionally, in the case of studies involving larvae of zebrafish up to 120 hpf, the approval of the Ethical Commission is not a prerequisite. This enables the undertaking of studies in this model that might otherwise be challenging or unfeasible in mammals [60].

This animal model could be successfully used as a model for the study of a variety of human diseases [61], including hematopoietic disorders [62], cardiovascular [63,64,65], metabolic [66], and kidney diseases [67,68], cancer [69,70,71], and central nervous system (CNS) disorders (e.g., alcohol disease, depression, anxiety, autism spectrum disorder, schizophrenia, Alzheimer’s disease) [72,73,74,75], bone diseases [76,77], etc. Furthermore, zebrafish exhibit a range of sophisticated behaviors that can be investigated in diverse settings. These include social behaviors (e.g., shoaling, aggression and mating, decision making in groups), motor responses, anxiety, learning, and memory. This renders them a valuable resource for the purposes of neurobehavioral testing [78,79,80]. Beyond that, the utilization of zebrafish in drug discovery and toxicology studies [81,82,83,84,85,86,87,88,89,90,91] is becoming increasingly prevalent. Their diminutive size and transparent embryos facilitate high-throughput screening of both natural and synthetic compounds, thus enabling the identification of potential therapeutic agents and the assessment of toxic effects in a living organism [45,83,92,93,94,95,96]. In order to gain a comprehensive understanding of biological processes and disease states, it is possible to utilize the zebrafish model in conjunction with genomics, proteomics and metabolomics [97,98,99]. This approach has the potential to enhance the precision of therapies and facilitate the development of personalized medicine.

While the use of zebrafish in research offers numerous advantages, including the development of new drugs, there are also some limitations that warrant consideration. Despite the existence of genetic similarities, the zebrafish remains a distant relative of the human species in comparison to other mammalian models, such as the mouse or rat [100]. This evolutionary divergence implies that some human-specific pathways and gene functions may be inadequately represented or exhibit substantial divergence in zebrafish [47]. The study of certain human diseases in this model is hindered by anatomical and physiological differences. This is particularly the case for diseases involving complex organs, such as the lungs, the prostate, and the mammalian immune system. The lack of lungs in zebrafish and the simplicity of their immune system make it challenging to model respiratory [101] and immune-related conditions [102,103,104]. Moreover, as an animal model, zebrafish exhibit notable differences in brain complexity compared to mammals and humans [105]. Additionally, the low level of inbreeding may contribute to increased variability in responses between animals, potentially complicating data analysis [106]. The metabolic processes of zebrafish differ from those of humans [49,107], which may restrict their use in pharmacokinetic and pharmacodynamic studies. It is possible that the absorption, distribution, metabolism, and excretion (ADME) of xenobiotics in zebrafish may not always mirror those observed in the human body [108]. Techniques for assessing ADME in zebrafish larvae are being developed but are still at an early stage [109,110,111]. This could have implications for the outcomes of drug discovery and toxicity testing.

In conclusion, the zebrafish model is an efficacious and adaptable tool in biomedical research, offering distinctive benefits that facilitate the investigation of genetic, developmental, and therapeutic aspects of diverse diseases. The application of this model is becoming increasingly prevalent, making a substantial contribution to the advancement of drug discovery and the comprehension of intricate biological systems. Nevertheless, it should be noted that this animal mode has certain limitations, which render zebrafish less appropriate for specific areas of inquiry, including those that require close human anatomical and physiological parallels, complex drug metabolism studies, or highly specific human disease research.

## 3. Studies of Coumarins and Coumarin-Based Compounds in Zebrafish Model

The use of zebrafish in research on coumarins and their derivatives is becoming increasingly common due to the distinctive advantages they offer as a model organism. This section delineates the contribution of zebrafish to studies on these compounds, particularly in the fields of toxicology and developmental biology, as well as in the evaluation of pharmacological efficacy, including anti-angiogenic activity and therapeutic properties in the treatment of CNS disorders.

### 3.1. Toxicological and Developmental Research

The zebrafish embryo is especially vulnerable to the effects of chemical compounds [57,112,113,114,115,116,117,118,119], making it an ideal model for evaluating the acute, chronic, and developmental toxicity of coumarins and their derivatives. The determination of the LC_50_ (lethal concentration 50%, lethal concentration of a compound at which 50% of the zebrafish embryos dies) is a common objective of acute toxicity studies in this species [120,121]. This parameter is frequently employed for the assessment of the acute toxicity of coumarins and their derivatives. These observations are employed in the calculation of the mortality rate of zebrafish exposed to varying concentrations of coumarins [122,123,124]. Moreover, the impact of coumarin or coumarin-based compounds on zebrafish is evaluated through the examination of discernible phenotypic characteristics, including hatching, edema, movement patterns, yolk sac utilization, heartbeat, body shape, swim bladder development, and otolith sac development using a stereomicroscope [122,123,124]. Another parameter employed in toxicological studies utilizing the zebrafish model is the EC_50_ (median effective concentration). The term is used to describe the concentration of a chemical compound that is capable of inducing an adverse effect in 50% of a specified population of organisms. In this context, the population in question is that of zebrafish. The term is also used in the context of describing the concentration of a compound that elicits a particular sub-lethal response. This parameter is frequently used for the purpose of evaluating the potency of coumarins in eliciting biological effects, including, for instance, developmental modifications or behavioral alterations, without necessarily leading to mortality [124]. It is of paramount importance in toxicological studies to determine both LC_50_ and EC_50_, as they offer invaluable insights into the safety margins and biological activity of coumarins, thereby facilitating the assessment of their associated risks and benefits.

The effects of coumarin on zebrafish embryos demonstrated a proclivity for malformation in the head and tail regions as well as growth delay. Notwithstanding the above, the LC_50_ and EC_50_ value was calculated to be 855 μM and 314 μM, respectively, which suggests that, in typical therapeutic conditions in humans, the teratogenic potential of coumarin may be relatively low when compared to warfarin (888 μM and 194 μM, respectively) [124]. Furthermore, a subsequent investigation into the impact of disparate coumarin derivatives on zebrafish embryos yielded no notable alterations in larval development and no discernible damage to their internal structures [122], indicating the potential for their further development as therapeutic agents.

The toxicity and teratogenic potential of coumarin compounds appear to be influenced by their structural properties. For example, 4-phenyl-hydroxycoumarins have been observed to exhibit different levels of embryo toxicity compared to basic coumarins [125]. The position and number of hydroxyl groups, as well as the presence of a phenyl group, have been found to affect both the cytotoxic activity of compounds and their preference for binding to protein kinases [125]. These findings serve to highlight the importance of considering the relationships between structure and activity when determining the toxicity and potential therapeutic applications of coumarin compounds. Moreover, coumarin has been identified as a hepatotoxic compound in zebrafish larvae, resulting in liver degeneration and discernible alterations in liver size. These findings were corroborated by both phenotypic assessments and gene expression analyses [126]. The study demonstrated that exposure to coumarin resulted in a significant reduction in the expression of liver-specific genes in zebrafish larvae compared to those treated in a control group. In particular, coumarin was found to have a detrimental effect on the expression of hepatocyte markers, including ceruloplasmin (CP), CYP3A65 (CYP3A ortholog), albumin-like (GC), liver fatty acid binding protein (FABP10a), and transferrin (TF) [126]. These findings underscore the hepatotoxic potential of coumarin in zebrafish larvae.

The objective of chronic toxicity tests is to evaluate the long-term effects of prolonged exposure to sub-lethal concentrations of compounds in zebrafish, with the goal of understanding the long-term consequences of exposure to potentially harmful substances. The tests are designed to evaluate the impact on a variety of biological parameters, including growth, organ function, reproductive capacity, and overall health, over an extended period of time, frequently spanning multiple weeks or even multiple generations [127,128,129,130].

The existing research on the chronic effects of coumarins using the zebrafish model is limited. Nevertheless, a study conducted by Blanc et al. [131] examined the multi- and transgenerational consequences of early-life exposure to 7-diethylamino-4-methylcoumarin in zebrafish. The study revealed that exposure to this coumarin did not result in significant multigenerational toxicity. This was assessed in relation to growth, fertility, behavior, and lipid metabolism in the F0 to F3 generations [131]. These findings suggest that, at least in the case of 7-diethylamino-4-methylcoumarin, chronic exposure during early development may not result in adverse effects in zebrafish across multiple generations. Nevertheless, further research is necessary to gain a comprehensive understanding of the long-term effects of various coumarins in zebrafish models.

Table 2 presents the findings of studies that evaluated the toxicity of coumarins and coumarin-based compounds.

### 3.2. Evaluation of Pharmacological Properties

#### 3.2.1. Anti-Angiogenic Activity

Angiogenesis (neovascularization) is a multifaceted process that is subject to strict regulatory control. All phases of angiogenesis, including endothelial cell migration, proliferation, and activation, are governed by a network of factors that either facilitate or impede the progression of this biological phenomenon [139]. It is a normal developmental process, but it has a particular significance in the formation of tumors. The new blood vessels support and nourish the neoplasms, enabling it to grow from benign to malignant, thereby increasing the likelihood of metastasis. Zebrafish possess a closed circulatory system, and the form of the developing vasculature, the processes used to assemble vessels, and the molecular mechanisms underlying vessel formation are strikingly similar to those observed in humans (for a review, see [139,140]). The formation of new blood vessels and the patterning of major vessels occurred in zebrafish embryos and larvae between 24 and 72 hpf [139,141]. The transparent nature of zebrafish embryos, coupled with their relatively simple genetics, make them an excellent model for real-time observation of blood vessel formation. Anti-angiogenic activity can be evaluated by assessing the inhibition of intersegmental vessel sprouting, alterations in caudal vein plexus formation, and a reduction in subintestinal vessel branching [142,143].

Chimote and co-workers (2013), in their comprehensive study, compared the effects of several anti-angiogenic agents in the zebrafish model [144]. The outcomes showed that the zebrafish model can be used to effectively stratify anti-angiogenic agents based on their mechanisms of action and to delineate biological activity based on the chemical structure [144]. The anti-angiogenic activity of coumarin in zebrafish models has recently emerged as a topic of growing interest, given its potential implications for cancer therapy, vascular diseases, and other angiogenesis-related conditions (for a review, see [145]). This part of article provides a concise overview of the current state of knowledge and research on this topic.

The objective of studies conducted by Anegundi and Pancharatna (2017) was to test the hypothesis that umbelliferon (7-hydroxycoumarin) and herniarin (7-methoxycoumarin) have anti-angiogenic activity in developing zebrafish embryos [132,146]. In order to trace the patterns of blood vessels, the pathways of blood flow, and the process of cell apoptosis, the entire preparation was stained with alkaline phosphatase, *O*-dianisidine, and acridine orange, respectively. Additionally, the presence of cardiac irregularities, notably changes in pericardial size and oedema, was recorded. Studies also investigated the impact of the treatment on heart rate, morphology, and mortality in zebrafish. The findings of this study suggest that umbelliferon has the capacity to disrupt the patterning of major blood vessels, such as intersegmental vessels (ISVs), dorsal aorta (DA), and posterior cardinal vein (PCV), which may subsequently result in a disruption to the normal flow of blood through them. These vascular impairments were accompanied by increased site-specific cellular apoptosis and reduced heart rates in a dose-dependent manner, indicating that 7-hydroxycoumarin and 7-methoxycoumarin anti-angiogenic effects are mediated through cellular apoptosis [132,146]. The 50% inhibitory concentration (IC50) for the inhibition of ISV growth was determined to be 2.65 mM [146] and 2.64 mM [132], respectively. It is notable that the observed effects were specific and dose-related, indicating that zebrafish embryos/larvae are a reliable and sensitive tool for defining and quantifying angiogenic activity.

The anti-angiogenic potential of coumarin derivatives has been the subject of recent investigation using zebrafish models. A series of coumarinyl 4-thiazolidinone derivatives (TZL1-TZL7) was synthesized by Bhat and Bhaskar (2024), and their anti-angiogenic activity was assessed using the zebrafish model [135]. Of the tested compounds, TZL2 exhibited the most pronounced anti-angiogenic effect, with an average vein count of 2.7, indicative of a marked reduction in angiogenesis. In addition, other derivatives, including TZL4, TZL5, and TZL6, exhibited significant anti-angiogenic effects [135]. These findings underscore the potential of these novel coumarin-based compounds for modulating angiogenesis and as potential anti-cancer agents.

Using zebrafish embryos, Huang et al. (2024) conducted a study to evaluate the angiogenesis-modulating effects of six newly synthesized coumarin derivatives (compounds 1–6) [136]. The outcomes showed that compound 2, which naturally occurs in some plants (i.e., *Plumbago zeylanica*, *Citrus grandis*, and *Naucleopsis caloneura*), is a promising anti-angiogenic agent with minimal toxicity (survival rates of 82.5–100%). The experiments employed transgenic zebrafish expressing green fluorescent protein in blood vessels (Tg(fli1:EGFP)) to investigate the impact of this agent on the development of intersegmental vessels, subintestinal veins, and caudal vein plexus remodeling. The findings revealed that compound 2 significantly disrupted these developmental processes [136]. In addition, molecular analysis conducted by the research team suggested that the likely mechanism underlying the anti-angiogenic activity of compound 2 may be mediated by the modulation of the gene expression of *nrp1a* [136], a co-receptor for the vascular endothelial growth factor receptor (VEGFR), which is involved in the positive regulation of cell migration and angiogenesis sprouting and is upstream of a positive effect on angiogenesis [147].

Majnooni et al. (2019), in their review, provided a critical analysis of currently available literature data on anti-angiogenic and anti-cancer mechanisms of natural and synthetic coumarins and highlight their use as a potential therapeutic strategy [145]. It is notable that the review in question refers to a single study, which was conducted using a zebrafish model. The study, conducted by Jung et al. (2009), demonstrated that decursin and decursinol angelate (natural coumarins derived from the *Angelica gigas* herb) markedly inhibited blood vessel development in transgenic zebrafish embryos at a concentration of 20 µM [148]. The study revealed the inhibition of VEGFR2 (a crucial VEGF receptor) and other VEGFR-related angiogenesis signaling pathways, such as phosphorylated extracellular signal-regulated kinase (p-ERK) and mitogen-activated protein kinase (MAPK), in addition to phosphorylated c-Jun N-terminal kinase (JNK) in endothelial cells [148].

In light of the cited research, it can be postulated that the zebrafish model is an invaluable tool for investigating the anti-angiogenic properties of coumarins, largely due to the transparency of its embryos, which affords real-time visualization of blood vessel formation. The vascular development of the zebrafish closely resembles that of humans, including similar molecular pathways, which makes it an excellent translational model. Additionally, zebrafish enable rapid, cost-effective screening of potential anti-angiogenic agents with minimal ethical concerns compared to mammalian models. Furthermore, studies using transgenic zebrafish expressing fluorescent markers in blood vessels enhance the precision of analyzing angiogenesis inhibition. The versatility and genetic simplicity of this model make it ideal for evaluating the therapeutic potential and mechanisms underlying the anti-angiogenic activity of coumarins and their derivatives.

#### 3.2.2. Central Nervous System Disorders

The zebrafish model offers distinctive advantages for the study of CNS disorders (including sleep–wake cycles, depression and anxiety, epilepsy, and neurodegenerative disorders) (for a review, see [85,105,149,150,151,152,153,154,155,156,157,158]) and the assessment of coumarin derivatives as prospective neuropsychopharmacological agents [4,159,160]. Anatomical and functional similarities between the zebrafish brain and the human CNS [153,161,162], including neurotransmitter systems, such as the dopaminergic, serotonergic, and glutamatergic pathways [162], make the zebrafish an excellent tool for investigating neurological mechanisms. The zebrafish larva displays a range of complex behaviors, including locomotion [163,164,165], anxiety-like responses [166], emotions [167,168,169], helplessness [170], social interaction [171], decision making [172], and cognitive functions [173]. These can be quantitatively assessed in order to evaluate the effects of coumarins on CNS function.

The potential of coumarins as neuroprotective agents has been demonstrated [159,160,174,175], primarily due to their anti-oxidant and anti-inflammatory activities (for a review, see [159]), which are crucial for the mitigation of oxidative damage and neuroinflammation observed in CNS disorders [176]. Zebrafish models are particularly well-suited to high-throughput screening of coumarin derivatives. Furthermore, transgenic zebrafish lines expressing fluorescent markers in specific CNS regions facilitate the precise tracking of neurodegenerative processes [177] and the assessment of the effects of coumarin treatments at the cellular level.

Zebrafish have emerged as a valuable model for investigating the mechanisms underlying the pathophysiology of and therapy for Alzheimer’s [157] and Parkinson’s disease [178,179,180]. The zebrafish have been genetically modified to express human amyloid precursor protein, which results in the formation of amyloid beta (Aβ) plaques, a defining characteristic of Alzheimer’s disease. Such an approach allows researchers to investigate the impact of Aβ accumulation on neuronal degeneration and behavioral traits, such as memory and learning deficits [157]. From a Parkinson’s disease research point of view, the zebrafish has a ventral diencephalon, which is thought to be similar to the mammalian substantia nigra. Two main types of zebrafish Parkinson’s models are used in experimental pharmacology, i.e., the neurotoxin-induced (1-methyl-4-phenyl-1,2,3,6-tetra-hydropyridine (MPTP), 6-hydroxydopamine (6-OHDA), paraquat, rotenone, and other neurotoxic agents) and the transgenic model (PTEN Induced Putative Kinase 1 (PINK1), Parkin RBR E3 Ubiquitin Protein Ligase (Parkin), DJ-1, α-synuclein, Parkinson’s disease protein 2, PD protein 7, leucine-rich repeat kinase 2 (LRRK2), and other gene mutations) [180,181]. Furthermore, zebrafish display sophisticated behaviors, including learning, memory, and social interactions, which can be employed to evaluate cognitive impairments associated with Alzheimer’s disease. Behavioral assays (for a review, see [182]), such as the novel object recognition test [183], the Y-maze test [183], the appetitive conditioning test, the inhibitory avoidance test [184,185], the novel tank test, and the locomotor activity test [186], facilitate the assessment of the efficacy of potential therapeutic compounds in the zebrafish model.

The utilization of these models permits the assessment of the potential of coumarins and their derivatives as therapeutic methodologies for these debilitating neurodegenerative disorders. Nevertheless, the potential of zebrafish models has yet to be fully explored in this context. In the course of searching the available databases for this review, no publication was identified in which the zebrafish model was employed to evaluate the activity of coumarins and coumarin-derived compounds as drug candidates for Alzheimer’s and Parkinson’s disease.

The zebrafish has additionally been identified as an invaluable model for the investigation of anti-epileptic and anti-convulsant properties of, for example, natural agents [187], due to its genetic, physiological, and pharmacological similarities to humans [49]. These include (1) significant parallels with the mammalian brain [188], particularly in terms of the neurotransmitter system [189,190] (such as glutamate and GABA), ion channels [191], and neural circuitry [192] that are implicated in the generation and propagation of seizures, (2) well-characterized, observable behavioral alterations that occur during epileptic episodes, inter alia hyperactivity, convulsions, and paroxysmal movements [193], which might be quantified, (3) the possibility of utilizing genetic manipulation techniques [193], which allows for the investigation of the specific role of genes in the pathophysiology of epilepsy and drug response, (4) the variety of methods that may be employed to induce seizures, including chemical induction with pentylenetetrazole (PTZ), kainic acid, or pilocarpine (for a review, see [193]), and (6) the fact that seizures in zebrafish respond to conventional anti-epileptic drugs, including diazepam [194] and valproic acid [195], which make it a validated model. The zebrafish model is becoming an increasingly popular choice for the study of natural compounds that may act as candidates for new anti-epileptic/anti-convulsant/anti-seizure medications.

The first systematic investigation of the anti-seizure activity of an array of coumarins, representing a diverse set of chemical structures, was conducted by Kozioł et al. (2021). They employed the PTZ seizure model in zebrafish larvae to assess the anti-epileptic activity of 18 coumarin derivatives, simple coumarin derivatives (daphnoretin, osthole, umbelliferone), linear furanocoumarins (8-geranyloxypsoralen, bergapten, byakangelicin, byakangelicol, imperatorin, notopterol, isoimperatorin, oxypeucedanin hydrate, oxypeucedanin, phellopterin and xanthotoxin), angular furanocoumarin (pimpinellin), dihydrofuranocoumarins (nodakenetin and nodakenin), and dihydropyranocoumarin (hyuganin C) [196]. The utilization of the zebrafish model permitted the authors to undertake a simultaneous behavioral and electrophysiological investigation, thereby facilitating the elucidation of the potential of different coumarins. In total, 7 out of 18 coumarin derivatives coumarin derivatives under investigation, namely oxypeucedanin, oxypeucedanin hydrate and notopterol (83–89% reduction of PTZ-induced elevations in power spectral density), nodakenetin (77%), hyuganin C (65%), daphnoretin (88%), and pimpinellin (81%), were identified as exhibiting the greatest potential as novel anti-convulsant agents. These agents demonstrated both anti-seizure and anti-epileptic efficacy in the locomotor activity assays [196]. These findings were further corroborated by electrophysiological assessments, which demonstrated that the majority of these coumarins markedly decreased PTZ-induced elevations in power spectral density, indicative of pronounced anti-epileptic activity.

Further research was conducted on the principal furanocoumarin constituent, halfordin, derived from the endemic plant Halfordia kendack [197]. In the initial phase of the experiments, the crude methanolic leaf extract of the plant, at concentrations of 75 and 100 μg/mL, was observed to markedly diminish seizure-like behavior in the PTZ seizure model in zebrafish. The subsequent isolation and testing of halfordin also demonstrated a notable reduction in convulsive-like behavior and the modulation of specific gene expressions associated with seizure activity when used in a 20 μM concentration [197].

These studies indicate the potential of coumarins as promising candidates for the development of new anti-seizure medications. The efficacy of these compounds in the zebrafish PTZ seizure model reinforces the value of this model in the initial stages of anti-seizure drug discovery.

A widely accepted model for neurobehavioral research is the study of anxiety behavior in zebrafish [198,199,200]. The ability to quantify a range of anxiety-like behaviors in zebrafish makes them an ideal subject for investigations into the effects of drugs, compounds or environmental stressors on anxiety [200,201]. The evaluation of anxiety in zebrafish employs a range of behavioral assays that are specifically designed to assess stress-related responses through the observation of behavioral patterns [202,203]. Most commonly employed tests in zebrafish to evaluate anxiety-like behavior are described in Table 3.

The study by Maciąg and colleagues (2020) represents the inaugural investigation into the impact of a naturally occurring metabolite, xanthotoxin, on anxiety-related behaviors. In this experiment, the level of anxiety in larval zebrafish was evaluated through the measurement of thigmotaxis [213]. The findings indicate that xanthotoxin exerts a reversed U-shaped influence on anxiety behaviors. Moreover, Maciąg et al. (2020) observed this phenomenon also in a murine model [213]. The parallel pattern of induced by xanthotoxin anxiolytic-like behavior in both animal models serves to demonstrate the predictive capacity of the zebrafish model for the investigation of anxiolytic properties of natural agents, including coumarins and coumarin-based compounds.

A significant contribution to this field was the research conducted by Widelski et al. (2021) offering the first comprehensive evaluations of anxiolytic-like activity of pyranocoumarins and devenyol [214]. The effect of a crude extract of *Seseli devenyense*, as well as isolated coumarins (devenyol, cis-khellactone, D-laserpitin, isolaserpitin and octanoyllomatin) on the thigmotaxis behavior of zebrafish larvae in response to changes in light/dark conditions were studied. The experiments were conducted in accordance with the methodology described by Schnörr et al. (2012) [206], as detailed in Table 3. The acute administration of crude extract of *Seseli devenyense* was found to generally enhance the locomotor activity of zebrafish larvae under light conditions. The ratios between the inner and outer arenas were altered in a manner that was more favorable to the inner arena, as evidenced by the time spent and distance moved within this region, indicating a reduction in thigmotaxis and an enhancement in anxiolytic activity, particularly with the higher concentrations (9, 12.5 and 25 μg/mL). Given the encouraging anxiolytic efficacy demonstrated by crude extract of *Seseli devenyense* in the zebrafish larvae model, the extract was employed for the isolation of coumarin compounds. Subsequent to this, these compounds were subjected to an anxiolytic efficacy assessment utilizing the zebrafish model. The administration of devenyol, D-laserpitin, isolaserpitin and octanoyllomatin resulted in a notable enhancement in the distance travelled by the larval zebrafish, as well as the time spent in the inner area during the dark phase of the experiment, in comparison to the light phase [214]. It is evident from these findings that these agents have the potential to be developed as therapeutic agents for anxiety disorders. Moreover, the findings indicate that the presence of a free -OH group at the C7 position of the coumarin structure may be pivotal for its anxiolytic efficacy, as evidenced by the activity of devenyol [199]. Subsequent studies investigating the anxiolytic effects of coumarins in zebrafish model have indicated that not only the presence of a free -OH group at C7 but also the absence of a -OCH_3_ group at C8 may represent crucial structural determinants underlying the observed pharmacological outcomes [215]. The objective of this study was to assess the anxiolytic efficacy of coumarins—officinalin, stenocarpin isobutyrate, and officinalin isobutyrate, derived from *Peucedanum luxurians* Tamamsch, using a five-day post-fertilization larval zebrafish. The anxiolytic activity of all tested coumarins was found to be significant. Of all compounds tested, officinalin and officinalin isobutyrate exhibited the most notable activity. Furthermore, the authors employed the zebrafish model to evaluate the impact of the investigated coumarins on the expression of genes associated with anxiety disorders. The results of these molecular research demonstrated that the observed behavioral effects were consistent with the gene expression profiles. The modulation was discernible in the expression of several genes, including those encoding *gaba-a, gaba-b, gal, htr1aa, htr1b, htr2b and penka*, in zebrafish exposed to officinalin and officinalin isobutyrate [215].

The utilization of the zebrafish model has been validated as an efficacious methodology for the screening of natural anxiolytic compounds, thereby providing a valuable instrument for future research in this domain. In addition, the aforementioned findings provided substantiation for the efficacy of the zebrafish model in assessing the impact of coumarin compounds on anxiety-related behaviors.

#### 3.2.3. Other Research

Conducted literature review also allowed to identify studies in which the zebrafish model was used to evaluate the anti-viral and anti-inflammatory activity of coumarins or coumarin-derived compounds.

A salient benefit of zebrafish lies in their capacity to serve as a rapid, transparent, and scalable system for high-throughput screening of potential anti-inflammatory and anti-viral compounds [82]. Utilizing live imaging and genetic tools, researchers can monitor immune cell behaviour, cytokine expression and viral responses in real time [216].

The translation of anti-viral and anti-inflammatory research from zebrafish to mammals relies on the high degree of evolutionary conservation in immune pathways. The presence in zebrafish of key components of the innate immune system, including Toll-like receptors (TLRs), cytokines (e.g., TNF-α, IL-6 and type I interferons) and signaling pathways (e.g., NF-κB and JAK-STAT), suggests their importance as a model system in anti-viral and anti-inflammatory studies in mammals [103,217]. This substantial similarity facilitates the study of the modulatory effects of compounds on immune pathways in zebrafish, prior to the employment of mammalian models. The utilization of zebrafish as a rapid, transparent and scalable system for high-throughput screening of potential anti-inflammatory and anti-viral compounds is predicated on the fact that it is a live subject in which the monitoring of immune cell behaviour, cytokine expression and viral responses can be accomplished in real time by the employment of live imaging and genetic tools [82,216].

However, it is important to note the limitations of this model. While zebrafish provide a good model for innate immune responses, their adaptive immune system develops later, meaning that early studies may not fully capture the long-term immunological memory or T-cell-mediated responses that are crucial in mammals [102]. Consequently, the validation of zebrafish findings in rodent models, which possess more intricate immune systems, is imperative.

Notwithstanding these limitations, zebrafish studies have a role in the drug discovery process. They facilitate the identification of promising candidates and refinement of dose prior to progression to mammalian models. This integrative approach serves to reduce the reliance on conventional early-stage mammalian testing and streamline the path to clinical research.

The pharmacological potential of coumarins has been demonstrated, particularly in the context of anti-inflammatory and anti-viral therapies. The anti-inflammatory properties of coumarins have been well documented, resulting from their ability to modulate the immune response, reduce oxidative stress, and inhibit the production of pro-inflammatory mediators, including cytokines, reactive oxygen species, and nitric oxide (for review see [218]). Also, the ability of coumarins to combat viral infections has been the subject of considerable interest, with research indicating that they can directly inhibit viral replication and enhance host anti-viral immune responses [219]. Their distinctive chemical configuration enables interaction with pivotal molecular pathways implicated in viral infections and inflammatory processes, rendering them promising candidates for pharmacological development. The following studies describe the utilization of the zebrafish model for the assessment of the aforementioned properties of coumarins.

The anti-viral properties of coumarins have been demonstrated in a zebrafish model by compelling evidence presented in research conducted by Liu et al. (2019, 2020). In one study, it was observed that the imidazole coumarin derivative (D5) inhibits the replication of the spring viremia of carp virus (SVCV), reduce viral titers, and enhance the immune response by up-regulating interferon-related genes in zebrafish [220,221]. Similarly, hydroxycoumarin (C10) was observed to significantly reduce mortality in SVCV-infected zebrafish (by 70%), to decrease viral replication, and to activate innate immune genes, thereby offering strong protection against infection. These studies highlight the potential of coumarins as a therapeutic agent for combating viral infections [220,221]. In turn, Yang et al. (2018) using a tail-cutting-induced zebrafish larvae model investigated the anti-inflammatory properties of bergapten, a coumarin derived from the roots of *Ficus hirta* [222]. On the basis of the obtained results, bergapten was highlighted as a natural compound capable to reduce neutrophil and macrophage migration to the injury site in zebrafish’s tail. Additionally, bergapten was observed to facilitate the clearance of these cells during the process of wound resolution. Moreover, this coumarin was able to reduce the levels of reactive oxygen species and nitric oxide, thereby demonstrating anti-inflammatory and pro-resolving effects [222]. The prevalence of lipid metabolism disorders, including obesity, is increasing at a global level [223,224], thereby necessitating the exploration of novel therapeutic agents. Also in this case, the zebrafish model is becoming an increasingly valuable tool for evaluating the efficacy of potential future treatments for lipid metabolism disorders [66]. Coumarins, renowned for their varied biological activities, have recently emerged as a potential avenue for managing lipid-related conditions [225]. A recent study by Zhao et al. (2024) investigated the lipid-lowering effects of a pair of coumarin-based enantiomers, i.e., (+)/(−)-Gerbeloid A, isolated from *Gerbera piloselloides* [226]. In a high-fat diet zebrafish model, both enantiomers were shown to significantly reduce triglyceride levels and total cholesterol. The observed effects were found to be dose-dependent, with no evidence of toxicity present at concentrations that were considered therapeutic [226]. The study highlights the potential of coumarins as therapeutic agents for the treatment of lipid metabolism disorders, such as obesity, and the value of the zebrafish model in studies of lipid-lowering and anti-obesity activity of coumarins and coumarin-based compounds.

## 4. Conclusions

In conclusion, the zebrafish model offers a valuable platform for advancing our understanding of the therapeutic potential of coumarins, particularly in the context of the central nervous system, metabolic disorders, inflammation, and viral infections. While the model’s genetic accessibility, physiological relevance, and scalability make it an indispensable tool for preclinical research, its full potential in exploring the broader biological activities of coumarins and their derivatives has yet to be fully realized. Specifically, while significant research has been conducted on their effects within the nervous system, studies on cancer, such as potential anti-angiogenic properties, remain less explored. To enhance the utility of zebrafish studies in preclinical drug discovery, it is essential to expand their scope to encompass a broader range of therapeutic areas, coupled with a more systematic approach to understanding coumarins’ diverse biological effects. There are still under-explored areas in the study of coumarin derivatives and their therapeutic applications using zebrafish models. For instance, the anti-seizure potential of various coumarin derivatives remains to be extensively charted. Furthermore, the investigation of the angiogenesis-modulating effects of certain coumarin derivatives is in its infancy.

Future research could focus on systematically analyzing a broader range of coumarin derivatives for their potential therapeutic effects, such as anti-convulsant, anti-inflammatory, and anti-cancer properties. The identification of specific knowledge gaps, such as the paucity of comprehensive studies on the long-term effects of these compounds and their mechanisms of action, would be valuable for advancing this field.

## Figures and Tables

**Table 2:** Outcomes of studies investigating the toxicological effects of coumarins and coumarin derivatives in the zebrafish model.

Tested Agents	Tested Period	Methods	Main Outcomes	Limitations	Ref.
Coumarin and warfarin (31.25 μM to 1500 μM)	0–72 hpf	Determination of LC_50_, EC_20_, EC_50_ and TI; evaluation (scoring) of lethality and teratogenic effects	The LC_50_ and EC_50_ values for coumarin are 855 μM and 314 μM, respectively. The LC_50_ and EC_50_ values for warfarin are 988 μM and 194 μM, respectively. Both coumarin and warfarin produce teratogenic and lethal effects in zebrafish embryos. In the case of coumarin, three endpoints are identified, namely malformation of the head and tail and growth delay. In contrast, malformation of the tail is the only one endpoint observed in the case of warfarin.	The study was limited to warfarin and coumarin, not exploring a wider range of substances. It also compared the effects of human therapeutic concentrations, rather than examining other potential exposure scenarios.	[124]
Herniarin (7-methoxycoumarin, 7MC) (1, 2, 3, 4, and 5 mM)	6–72 hpf	Evaluation of cardiac anomalies/heart rates, cellular apoptosis, morphological deformities, and mortality rates; determination of LC_50_	The exposure of embryos to concentrations of 7MC in excess of 4 mM resulted in a reduction in heart rate, distortion of the tail, an increase in cellular apoptosis, and an elevated mortality rate.	Long-term effects of herniarin were not examined, and the study was conducted using a limited concentration range, overlooking the activity at sub-lethal doses.	[132]
4-phenyl hydroxycoumarins: 7C, 5,7C, and 7,8C (1, 10, and 100 μg/mL)	0–96 hpf	Evaluation of developmental effects and determination of TI; determination of LC_50_ and EC_50_	LC_50_ are lower for 7,8C and 5,7C compared to coumarin. 5,7C and 7,8C are more embryotoxic while 7C is less toxic in comparison to coumarin. Observed teratogenic and lethal effects of 4-phenyl hydroxycoumarins are concentration dependent.	There is further need to explore pharmacokinetic properties of 4-phenyl hydroxycoumarins, especially the mechanism of cAMP-dependent protein kinase inhibition, perhaps on other model organisms.	[125]
4-phenyl hydroxycoumarins: 7C and 5,7C (1, 5, 10, 25, and 50 μg/mL)	24–72 hpf	Determination of anti-melanogenic effect	The compounds 7C and 5,7C effectively reduce pigmentation in zebrafish embryos at a low dose of 5 µg/mL, and no toxicity is observed at higher doses. The 7C has a more favorable toxicity profile in comparison to the commonly used depigmenting agents, namely hydroquinone and kojic acid. This suggests that they may be more suitable candidates for use as skin-whitening agents. The anti-melanogenic effect of the 7C is likely attributable to the inhibition of the enzyme tyrosinase, as evidenced by computational molecular modeling studies.	The study does not report toxicity. The authors have exclusively concentrated on the anti-melanogenic effect of tested agents.	[133]
Fluorescent coumarin derivatives (1 μg/mL)	0–120 hpf	Imaging of retinal development	Four coumarin derivatives were identified as potential stains and imaging agents for zebrafish retinal cells in vivo. The coumarin derivatives may be employed to evaluate retinal development and identify morphological irregularities, in addition to serving as a counterstain for transgenic zebrafish expressing fluorescent proteins in particular retinal cell types.	While the study does not report toxicity, the long-term effects of coumarin derivatives on zebrafish development and retinal function remain unknown. The study also does not extensively compare the performance of coumarin derivatives with other established fluorescent dyes for retinal imaging.	[134]
Hybrid compounds incorporating 6- and 7-substituted coumarins (12.5 μM to 3 mM)	0–120 hpf	Determination of LC_50_; phenotypic analysis (hatching, oedema, movement pattern, yolk sack utilization, heartbeat, body shape, swim bladder development, and otolith sac development); swim pattern analysis; histological studies	The compounds were observed to exhibit minimal toxicity. The analysis revealed that none of the compounds induced discernible alterations in the developing zebrafish larvae, nor did they exhibit any indications of damage to internal tissues. The larvae exposed to the tested compounds did not display any abnormal or ataxic movement patterns.	The study was conducted over a short exposure period, without acknowledging long-term effects. It also lacked the comprehensive histopathological analysis and other toxicological parameters, such as organ damage or immune system effects.	[122]
Coumarinyl 4-thiazolidinone derivatives (TZL1-TZL7) (1, 3, 5, and 10 ppm)	0–120 hpf	Assessment of developmental toxicity, cardiotoxicity, neurotoxicity, and hepatotoxicity	Notable abnormalities or alterations in the embryos’ development were observed, indicating the potential for toxicity of coumarinyl 4-thiazolidinone derivatives at varying concentrations.	The study noted that higher concentrations caused significant adverse effects. It also lacks the information about the long-term efficacy of the compounds.	[135]
Coumarin derivates (1, 5, 10, and 15 ppm)	12–72 hpf	Survival rate analysis	Compounds 1, 2 (naturally occur in some plants, e.g., *Plumbago zeylanica, Citrus grandis, Naucleopsis caloneura*) and 4 (survival rates: 100%, 82.5–100%, and 100%, respectively) are less toxic than compounds 3, 5, and 6.	The study was limited to zebrafish and chick models, without further investigation in a mammal model. It also only covered gene expression changes, not directly measuring angiogenesis.	[136]
*p*-Coumaric acid (50 μM and 100 μM)	10–48 hpf	Determination of anti-melanogenic effect	The exposure of developing zebrafish embryos to *p*-coumaric acid results in a significant reduction in pigmentation. In comparison to phenylthiourea (200 μM), a known melanogenic inhibitor, *p*-coumaric acid has been demonstrated to be more effective at inhibiting pigmentation. *p*-Coumaric acid has been shown to exhibit a stronger binding affinity to the tyrosinase enzyme than phenylthiourea.	There is no information about the long-term toxicity of *p*-coumaric acid. The study also did not explore in depth the effects on organ development and reproductive health.	[137]
Mucorisocoumarins A, B, and C (10, 20, 30, and 50 μM)	0–48 hpf	Developmental toxicity test	Mucorisocoumarin C at a dose 20 μM causes defects in embryos (i.e., abnormal brain shape and a short tail). Higher concentrations led to more severe defects and embryo death. None of the other compounds showed any toxicity.	The study has limited structural data—some of the structures were not confirmed with X-ray crystallography. The long-term effects were also not explored.	[138]

5,7C, 5,7-dihydroxy-4-phenylcoumarin; 7C, 7-hydroxy-4-phenylcoumarin; 7,8C, 7,8-dihydroxy-4-phenylcoumarin; 7MC, 7-methoxycoumarin; dpf, day post fertilization; EC_20_, 20% effect concentration; EC_50_, median effective concentration; hpf, hours per fertilization; LC_50_, 50% lethal concentration; TI, teratogenicity index.

**Table 3:** Commonly employed behavioral tests in zebrafish for evaluating anxiety-like behavior.

Behavioral Test	Age of Zebrafish	Setup	Behavioral Endpoints	Interpretation	Ref.
Video-tracking analysis	Larvae of zebrafish	Larval zebrafish are observed in 6-well plate (1 zebrafish/well).	The angular velocity and turn angle, thigmotaxis (preference for the tank walls), total distance traveled during the test, time spent in predefined zones, duration of immobility.	An increase in the time spent in the bottom zone or dark areas, a higher freezing response, or a reduction in exploration and a decrease in angular velocity and turn angle are indicative of anxiety-like behavior. Anxiolytic compounds reduce these anxiety markers, thereby promoting exploration and reducing immobility.	[200,204]
Acoustic startle response test	Larvae of zebrafish	Larval zebrafish are observed in 96-well plate (1 zebrafish/well). Fish are presented with a flat spectrum “white noise” (1–10,000 Hz) stimulus at 20 dB re·1 ms^−2^.	The interval between the onset of the stimulus and the initiation of the motor response, the intensity of the C-movement, a reduction in the magnitude over the course of repeated trials.	An increased startle response indicates a heightened state of anxiety or stress. Anxiolytic drugs (e.g., benzodiazepines) can reduce acoustic startle response magnitude.	[200,205]
Thigmotaxis measure	Larvae of zebrafish	Larval zebrafish are observed in 24-well plate (1 zebrafish/well). The experimental procedure is performed in two steps: acclimatization (0–6 min, light on) and visual motor challenge (7–10 min, light off).	Swimming activity: general locomotor activity, thigmotaxis (distance moved and time spent in outer and inner zone).	Fish that are experiencing anxiety tend to exhibit a preference for swimming in close proximity to the tank walls (thigmotaxis), as opposed to exploring the central area. The anxiolytic effects are indicated by an increased exploration of the central area.	[200,206]
Light-dark preference test	Adult zebrafish	The tank is subdivided into two distinct zones, characterized by varying levels of illumination. One area of the tank is opaque black, with a partition situated in the center and a 2 cm height leak at the base, allowing the fish to pass through.	Time spent in the dark and light area.	Anxiolytic compounds enhance the time spent in the light area.	[200,207,208]
Novel tank diving test	Adult zebrafish	The zebrafish are placed into an entirely novel environment—in a new tank. The novel tank is comprised of two distinct arenas, delineated by a baseline situated at the center of the tank. This baseline serves to partition the tank into upper and lower arenas.	Time spent in the upper/lower zone, erratic movements, time duration of freezing and swimming toward the bottom.	The typical effect of anxiolytic compounds is a reduction in the time spent at the bottom of the tank and an increase in exploration of the upper zones.	[200,202,209]
Predator exposure test	Adult zebrafish	The tank incorporates a vertical plane, the function of which is to separate the predator from the test fish.	Freezing time, erratic swimming, time spent in the greatest distance from the predator, thigmotaxis.	Fish exhibiting a normal anxiety response demonstrate increased freezing, erratic swimming, and avoidance of the predator zone upon exposure to predator cues. The administration of anxiolytics was associated with a reduction in freezing and avoidance behaviors, an increase in exploration, and a decrease in thigmotaxis.	[204,210]
Shoaling test	Adult zebrafish	The observation tank is constructed with plexiglass walls and has dimensions of 17 cm × 17 cm × 5.75 cm. The tank holds 250 mL of water per fish (10 fish/tank).	Reduction in shoaling behavior, characterized by fish swimming in closer proximity to one another.	The reduction in shoaling distances indicates an elevated level of anxiety.	[200,203]
Social interaction test	Adult zebrafish	The observation tank is divided into three zones: nonsocial, social zone, and zebrafish room.	Frequency and duration of zebrafish activity in social zone.	The fish with higher level of anxiety exhibits reduced social interaction, avoidance of conspecifics, and displays freezing or erratic movements.	[200,208,211]
Social preference test	Adult zebrafish	The tank is divided into three chambers (five zones): a, e—social stimulus zone; b, d—area of social preference; c—area of no social preference.	Time spent near stimulus zone, number of entries into stimulus zone, distance maintained from the stimulus fish, freezing or avoidance.	Fish with a low level of anxiety tend to spend more time in the stimulus zone and frequently interact with conspecifics. A reduction in the time spent in the stimulus zone, along with evidence of avoidance or freezing, may indicate the presence heightened anxiety, or stress.	[200,212]

## Data Availability

No new data were created or analyzed in this study. Data sharing is not applicable to this article.
